# Influence of urban and agricultural land use on trace metal contamination in the Rio do Campo watershed, Paraná, Brazil

**DOI:** 10.1007/s10661-026-15150-2

**Published:** 2026-03-22

**Authors:** Taila Lorena De Souza, Oscar Vicente Quinonez Fernandez, Jefferson de Queiroz Crispim

**Affiliations:** 1 Departamento de Geografia, Universidade Estadual de Maringá, Maringá, Paraná, Brazil; 2 Departamento de Geografia, Universidade Do Oeste Do Paraná, Marechal Cândido Rondon, Paraná, Brazil; 3 Departamento de Geografia, Universidade Estadual Do Paraná, Campo Mourão, Paraná, Brazil

**Keywords:** Trace metals, Physicochemical parameters, Land-use change, Subtropical watershed, Paraná, Brazil

## Abstract

This study evaluated the water quality of the Rio do Campo watershed, located in the municipality of Campo Mourão, Paraná State, through the analysis of physicochemical parameters and the quantification of total concentrations of trace metals (Cu, Zn, Mn, and Fe) using atomic spectrometry procedures following the Standard Methods for the Examination of Water and Wastewater (24th ed.). Sampling was carried out at five points with different land uses and occupations, in urban and rural areas, over a 6-month period. Data were summarized using median values and analyzed using non-parametric statistical approaches. The results indicated statistically significant differences between rural and urban areas, with higher trace metal concentrations and turbidity at urban sites, attributed to the input of effluents and increased surface runoff. Parameters such as pH, BOD, COD, and DO exhibited variation consistent with the land-use gradient. Principal component analysis revealed overlap among rural, urban, and intermediate sites, indicating the presence of a continuous environmental gradient along the basin. All concentrations remained within the limits established by CONAMA Resolution No. 357/(Tundisi 2005). Nevertheless, the observed spatial distribution reinforces that the system is sensitive to local anthropogenic pressures, particularly in urbanized sections, highlighting the need for continuous monitoring to prevent potential future changes in water quality.

**Supplementary Information:**

The online version contains supplementary material available at 10.1007/s10661-026-15150-2.

## Introduction

Small watersheds, such as the Rio do Campo basin, located in the municipality of Campo Mourão, Paraná State, southern Brazil, are highly sensitive to changes in land use and land cover because of their physical characteristics and susceptibility to anthropogenic disturbances (Poleto, (Poleto 2012)).

The metals analyzed in this study, copper (Cu), zinc (Zn), manganese (Mn), and iron (Fe), have environmental relevance because of their association with regional processes. Cu and Zn are present in agrochemical inputs commonly used in the municipality’s predominant agricultural activities and may reach aquatic environments through surface runoff (Barros et al., 2022; Oliveira Silva  et al., 2020). Mn and Fe, in turn, are linked to local geology and the weathering processes of Red Latosols, serving as indicators of erosion, sediment transport, and leaching during periods of greater hydrosedimentary instability (Almeida et al., 2019).

The assessment of water quality parameters such as biochemical oxygen demand (BOD), chemical oxygen demand (COD), and turbidity allows the identification of soil particle input and the effects of organic matter and organic load (Tundisi & Matsumura-Tundisi, 2009). In urban areas, increases in BOD and COD may be related to the discharge of untreated or inadequately treated domestic sewage or effluents, while turbidity may indicate erosive processes or soil particle transport, especially in areas with unplanned land occupation (Von Sperling, 1996; Tundisi & Matsumura-Tundisi, 2009).

Despite the environmental importance of the Rio do Campo basin, up-to-date studies integrating physicochemical parameters and trace metals across different land-use zones are still scarce. This scientific gap limits the understanding of both anthropogenic and natural pressures acting on the basin, as well as the identification of stretches susceptible to environmental degradation. Updated data are essential to support management actions, urban planning, and the conservation of water resources, particularly in light of the intensification of agricultural activities and the urban expansion of the municipality.

We hypothesized that the urbanized sections of the sub-basin exhibit higher metal concentrations and greater limnological instability compared to rural stretches, due to effluent discharge, soil impermeabilization, and intensified surface runoff. The general objective was to evaluate water quality in the Rio do Campo sub-basin, considering spatial variations and relating physicochemical patterns and trace metal concentrations to land use and land cover.

## Materials and methods

### Study area

**Map 1:**
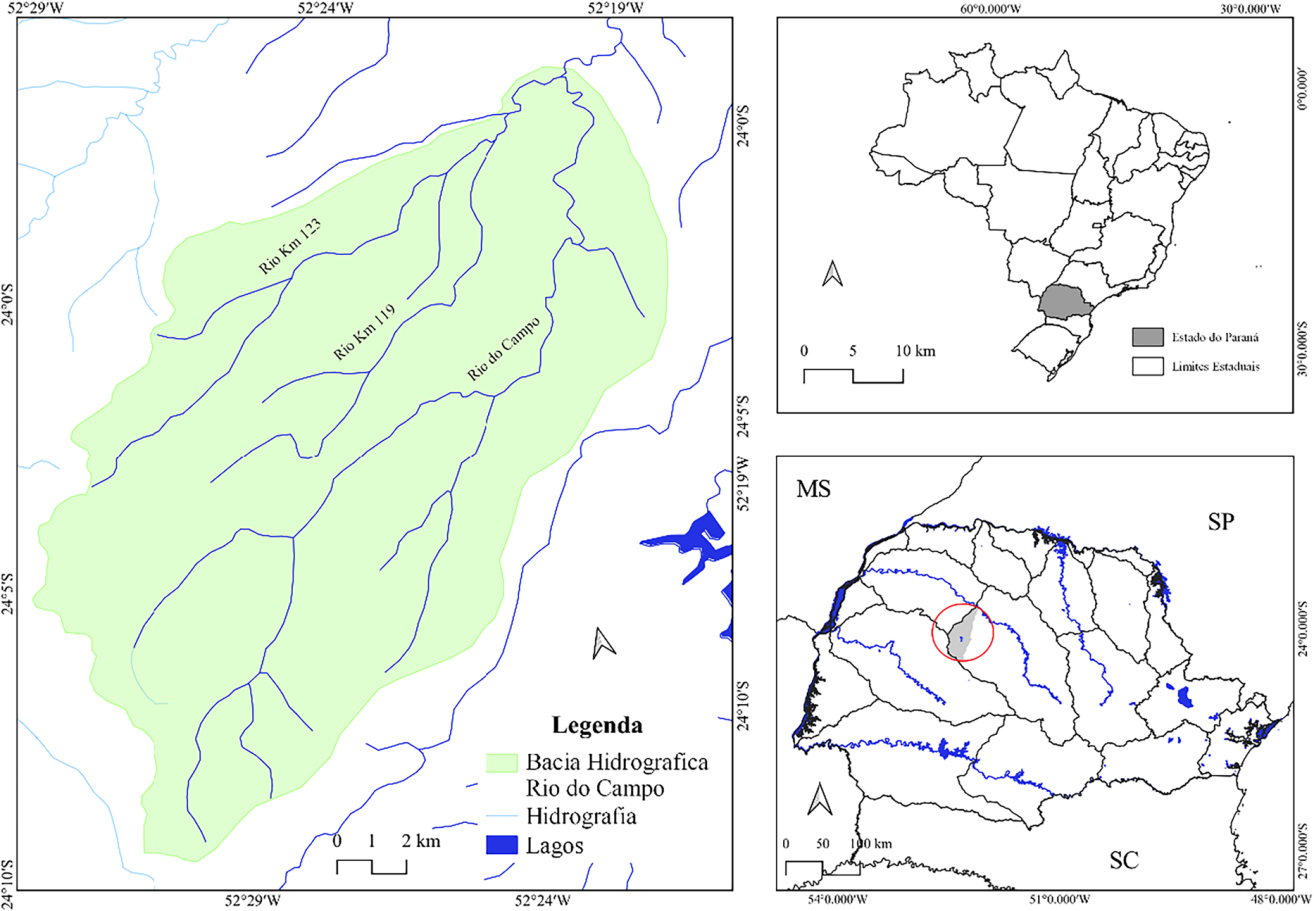
Location of the study area. Source: IBGE, 2020; organization: the authors, 2024

This study was conducted in the Rio do Campo watershed (Map 1), located in the municipality of Campo Mourão, in the Central-Western Paraná Mesoregion, Brazil (Maack, 2012). The municipality has an average altitude of 550 m and is situated in the middle sector between the Ivaí and Piquiri rivers (MINEROPAR, 2006). Map 1Location of the study area. Source: IBGE, 2020; organization: the authors, 2024Map 1Location of the study area. Source: IBGE, 2020; organization: the authors, 2024Map 1Location of the study area. Source: IBGE, 2020; organization: the authors, 2024

According to Strahler’s (1952) fluvial hierarchy, the watershed is classified as fourth order, comprising 71 channels. This study evaluated the main course of the Rio do Campo and its tributaries, the Km 119 and Km 123 rivers.

The Rio do Campo stretches 54 km, is the main left-bank tributary of the Mourão River, and flows through both urban and rural areas of the municipality. The Km 119 River extends 16.43 km and flows predominantly through the urban area. The Km 123 River, with a length of 16.66 km, is located in the northern part of the municipality in a predominantly rural area and is classified as third order (IAT, 2020).

The watershed contains clayey soils of basaltic origin, including Dystroferric Red Latosols and Dystrophic Red Argisols of sandy/medium texture, composed of reddish sandstones of medium granulometry derived from the Goio-Erê Formation (Fernandes & Coimbra, 1994).

The predominant climate in the Rio do Campo basin, according to the Köppen classification, is humid subtropical (Cfa), with temperatures ranging from 18 to 22 °C (Caviglione et al., 2000).

**Map 2:**
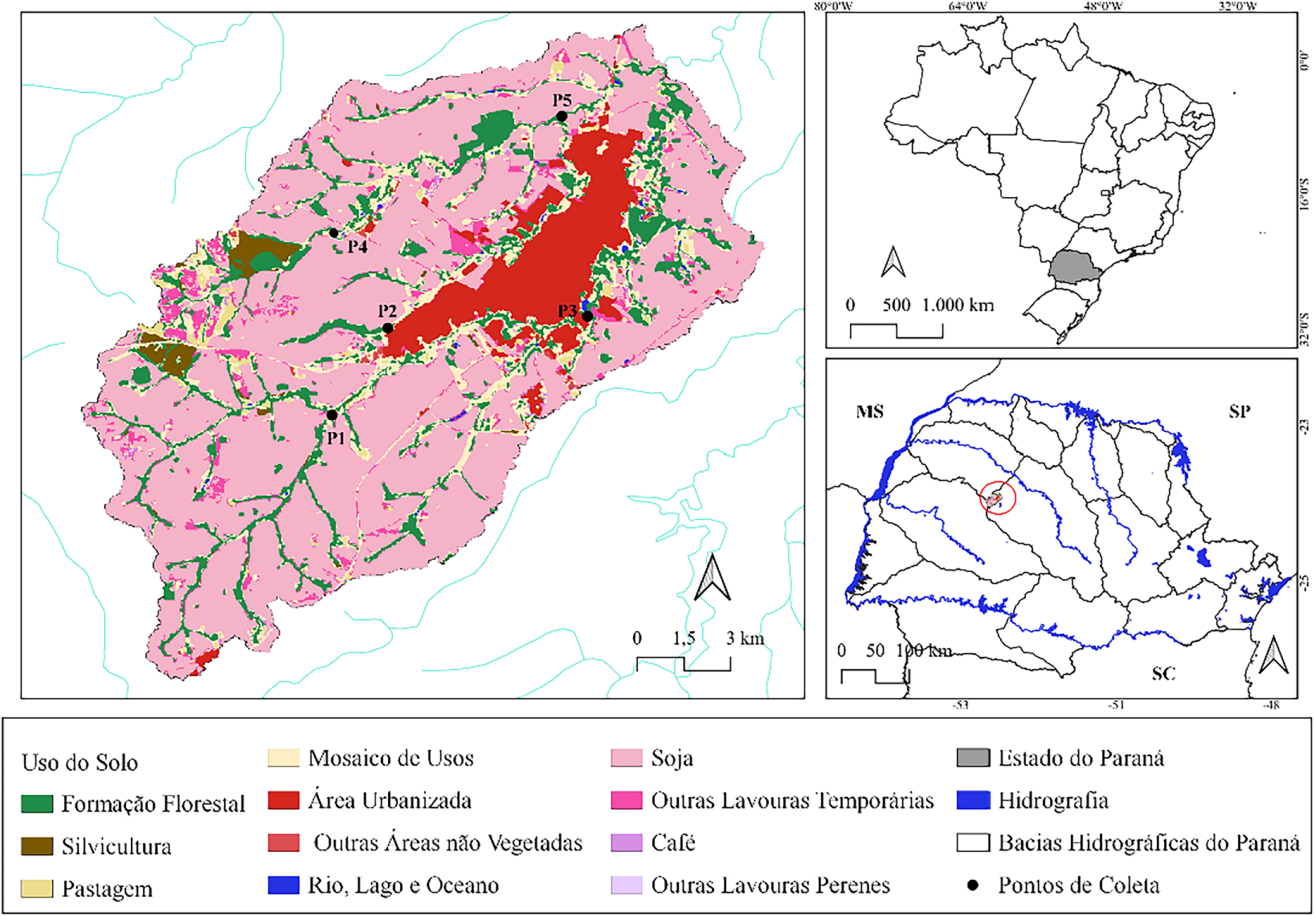
Land use in the study area. Source: MapBiomas, 2020; organization: the authors, 2024

Land use and land cover (Map 2) in the area encompassing the main watercourses of the Rio do Campo watershed can be classified into two main categories: urban use and agricultural use, the latter consisting primarily of soybean, corn, and wheat crops (Silva & Gasparetto, 2016).

Point 2 (24°3′22.44″S, 52°25′30.44″W) and P5 (23°59′21.62″S, 52°22′12.85″W) are located along the Km 119 River. In this region, land use is classified as 37% agricultural, 13% vegetation cover, 7% exposed soil, and predominantly urban (43%), especially along the right bank (MAPBIOMAS, 2020).

Point 5 is located downstream from the municipal wastewater treatment plant (WTP). Thus, considering both land use and the direct influence of a point-source discharge, P5 was classified as an intermediate point, not fitting strictly into the “rural” or “urban” categories. Although situated in an agricultural matrix, its downstream position relative to the WTP and the high proportion of urban area in the sub-basin justify its classification in a distinct group, representing mixed environments or areas influenced by treated effluent.

Point 1 (24°5′1.34″S, 52°26′33.88″W) and P3 (24°3′8.86″S, 52°21′43.80″W) are located along the Rio do Campo. The surrounding area comprises 75% agricultural land, 16% vegetation (mostly permanent preservation areas—PPAs), 8.7% urban area, and 0.3% exposed soil or pasture. This river is one of the main watercourses of the municipality (MAPBIOMAS, 2020). Point 1, upstream, is near the municipal water supply intake. Point 3 is located in a lake area created for recreational purposes within the urban area (MAPBIOMAS, 2020).

Point 4 (24°1′34.45″S, 52°26′32.00″W) is situated along the Km 123 River, within an agricultural cultivation area. This river stretch has minimal contact with urban areas and contains the largest extent of vegetation along its PPAs. Land-use classification in this region indicates 69% agricultural land, 26% vegetation, 4% urban area, and 2% exposed soil (MAPBIOMAS, 2020).

The selection of these five points ensures spatial and environmental representativeness within the Rio do Campo watershed. The points are distributed along the main watercourses (Rio do Campo, Km 119, and Km 123), covering upstream–downstream gradients, different land-use contexts (predominantly agricultural, urban, and intermediate), and areas subjected to varying anthropogenic pressures, such as the presence of a WTP and an urban lake. This combination of less impacted stretches, agricultural areas, and urban sectors allows for the capture of hydrological and environmental variability within the watershed, ensuring that the selected points are representative of the actual conditions of the studied fluvial system.

### Sampling

Five fixed points were sampled along the river (P1–P5). Each point was sampled during six consecutive monthly campaigns, conducted between June and December 2020, totaling 30 samples (5 points × 6 months). The campaigns encompassed the dry season (June–August) and the beginning of the rainy season (September–December) in the Rio do Campo watershed. During this period, the mean precipitation was 100.2 mm (AGUASPARANÁ, 2020), lower than the interannual mean for the same interval, which was 117.2 mm (Szapak et al., 2021). In each campaign, one sample was collected per point, and laboratory analyses were performed with analytical replication as described in the QA/QC section.

All sampling activities were conducted during the morning period (between 08:00 and 11:00 a.m.). Morning sampling is recommended for hydrological monitoring because it minimizes diel fluctuations in dissolved oxygen, temperature, and pH, ensuring more stable and comparable physicochemical conditions among sites.

The selection of points was based on satellite image analysis and field visits, prioritizing regions that encompassed different land uses and land covers, as well as representative physicochemical and hydrological characteristics of the watershed. Sampling points were established in both upstream and downstream sections.

Water samples were collected in situ in duplicate, following the guidelines of the National Guide for the Collection and Preservation of Samples: water, sediment, aquatic communities, and liquid effluents ( CETESB, 2015), established by the Environmental Company of the State of São Paulo (CETESB), and ANA (2011).

In the field, samples were collected in 500-mL polyethylene bottles previously washed with neutral detergent and rinsed with deionized water. Water was collected at a depth between 0 and 30 cm below the surface. After collection, bottles intended for metal analysis were acidified with ultrapure HNO_3_ to pH < 2, following the recommendations of the National Guide for Sample Collection and Preservation (CETESB & ANA, 2011). All samples were kept in insulated coolers at 4 °C and transported to the laboratory on the same day. To avoid cross-contamination, all bottles were pre-cleaned and handled with disposable gloves during sampling. For physicochemical parameters, samples were analyzed without dilution or additional treatments, following the procedures specified in the corresponding APHA, ABNT, and ISO standards.

Analyses were performed by a laboratory accredited by ABNT NBR ISO/IEC 17025 (CRL 1684) and participating in Embrapa’s Laboratory Quality Analysis Program for Fertility (PQALF). Procedures from the Standard Methods for the Examination of Water and Wastewater (24th ed.) and ABNT standards were followed. Metals were determined using an inductively coupled plasma mass spectrometer (ICP-MS) model 820-MS (Bruker).

Dissolved oxygen (DO) was determined using the iodometric method, as described in Baird et al., (2017) . pH was measured using the electrometric method (ABNT, 1988; Baird et al., 2017). BOD was determined through the respirometric method (ISO, 1999), while COD was measured using the colorimetric method (NBR 10705, 1989;  Baird et al., 2017 ). Turbidity was analyzed using the nephelometric method (Baird et al., 2017). For the determination of suspended solids, the Imhoff cone test was employed (Baird et al., 2017).

The results obtained were interpreted based on the standards established by CONAMA Resolution No. 357/2005, which defines conditions and quality standards for limnological parameters and dissolved metal concentrations in water.

The selection of physicochemical parameters and metals reflects the specific environmental characteristics of the Rio do Campo watershed. The region contains soils predominantly derived from basaltic rocks of the Serra Geral Formation, including Dystroferric Red Latosols and Red Argisols, which are naturally rich in iron and manganese oxides, justifying the monitoring of these elements in surface waters. Additionally, areas with intensive agricultural use may contribute to the availability of micronutrients such as Cu and Zn, which are commonly associated with fertilizers and soil amendments.

The mineralogical composition of these soils, combined with the presence of urban environments with potential inputs of domestic effluents, reinforces the importance of monitoring physicochemical parameters sensitive to anthropogenic influence, such as turbidity, BOD, COD, suspended solids, and pH. Thus, the methods employed allow the assessment of both natural contributions from the soils and local geology, as well as anthropogenic influences resulting from land use and land cover.

### Statistical analysis

Data were analyzed using RStudio software (version 4.4). The variables (Cu, Fe, Mn, Zn, pH, DO, turbidity, BOD, and COD) were compared according to sampling points, grouped into rural and urban categories.

Data were summarized using median and interquartile range (Q1–Q3) due to non-normal distribution and absence of laboratory-provided detection and quantification limits. Comparisons between rural and urban areas were performed using the paired Wilcoxon signed-rank test, considering months as paired blocks. Comparisons among sampling points were carried out using the Friedman test, with months treated as blocking factors, followed by the Nemenyi post hoc test when significant. Comparative statistical analysis was carried out only between the rural and urban categories, since the intermediate point (P5) does not belong to either paired class. For this reason, P5 was not included in significance tests and was used solely for descriptive purposes (median and interquartile range).

To investigate correlation patterns among variables across sampling points, a principal component analysis (PCA) was performed using standardized data with mean-centering (center = TRUE) and variance scaling (scale = TRUE) to eliminate the influence of differing measurement units and ensure comparability among variables. The importance of each component was evaluated based on eigenvalues and explained variance, represented in a scree plot. Ecological interpretation was grounded on the factor loadings and the distribution of samples in the biplot, distinguishing land-use categories (rural, urban, intermediate).

Additionally, a Pearson correlation matrix was calculated for metals and physicochemical variables to identify relevant linear associations among parameters. A significance level of *α* = 0.05 was adopted for all statistical tests.

### Quality assurance and quality control (QA/QC)

Physicochemical and metal analyses were performed in a laboratory accredited according to ABNT NBR ISO/IEC 17025 (CRL 1684), following established quality assurance and quality control (QA/QC) procedures.

Equipment calibration was carried out using certified standards. The pH meter was calibrated with buffer solutions of pH 4.0, 7.0, and 10.0. Turbidity was calibrated using formazin standards (0, 20, 100, and 800 NTU). For COD analyses, calibration curves were prepared from potassium dichromate standard solutions, according to NBR 10705 (1989). The equipment used for the remaining physicochemical determinations followed calibration procedures specified in their corresponding standards.

Laboratory blanks (reagents without sample) and analytical blanks were used to detect possible contamination during sample preparation, digestion, and analysis. All determinations were performed in analytical duplicates and, when applicable, digestion replicates were included to ensure the reproducibility of measurements and the precision of results.

Analytical accuracy was verified using traceable analytical standards, following the acceptance criteria established by ISO/IEC 17025. Calibration curves were considered valid when they presented a coefficient of determination of *R*^2^ ≥ 0.995. Certified reference materials NIST SRM 1640a and NIST SRM 1641 d were used, with a recovery rate of 102%.

## Results and discussion

### Results and discussion

**Fig. 1:**
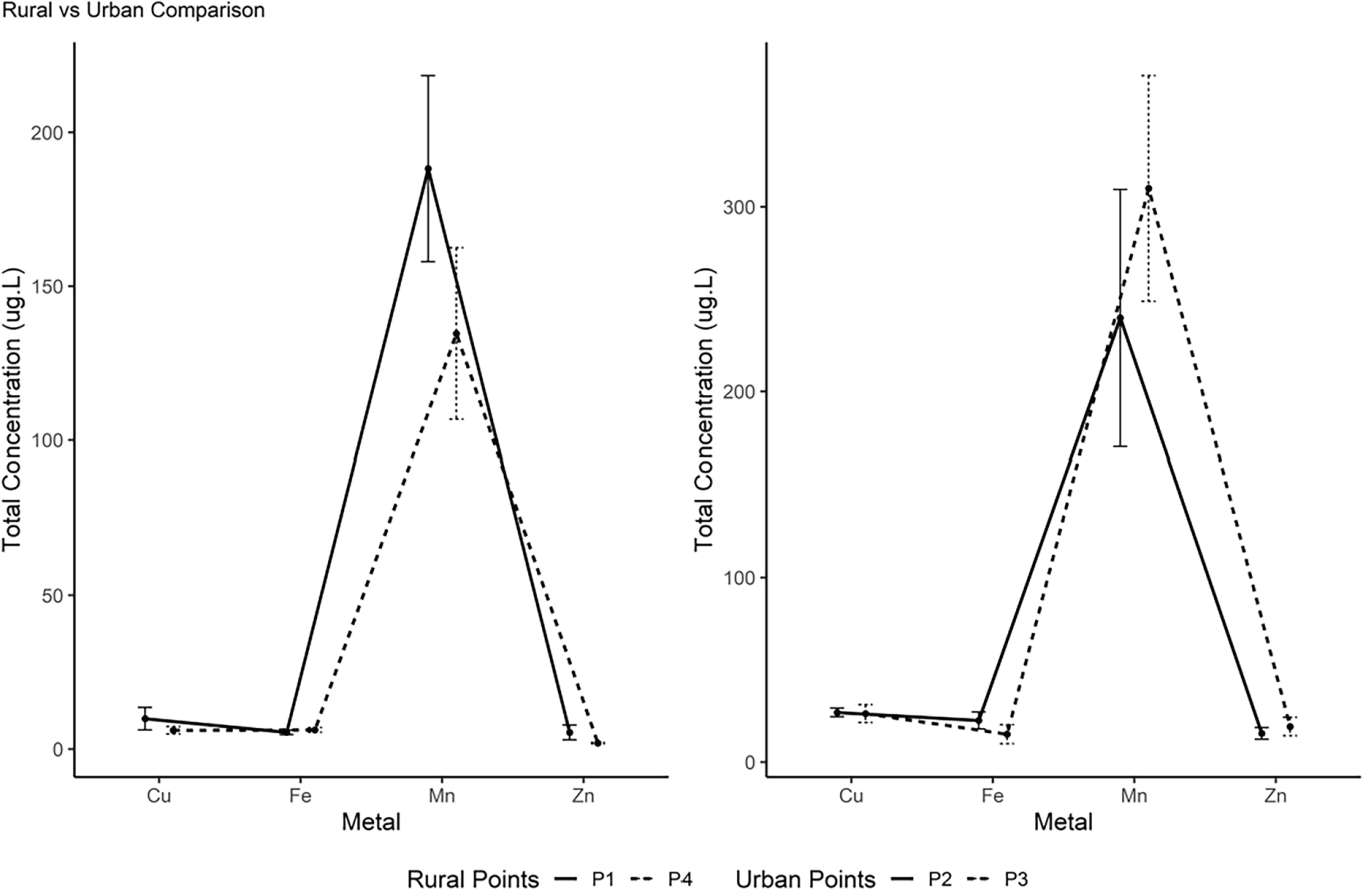
Comparison of median concentrations (Q1–Q3) of Cu, Fe, Mn, and Zn between rural (P1 and P4) and urban (P2 and P3) points

When the locations were compared, the urban area showed concentrations of Cu, Mn, Zn, Fe, turbidity (*p* = 0.0225), and pH (*p* = 0.0346). Mn was the metal with the highest concentration in both areas (Fig. 1). All metal concentrations remained within the limits established by CONAMA Resolution No. 357 of March 17, 2005.

The intermediate zone exhibited values similar to those found in the urban area (Table 1). Among the limnological variables, turbidity was also higher in the urban area (*p* = 0.02), while pH showed an opposite pattern, with higher values in the rural zone (*p* = 0.007). DO, BOD, and COD did not show significant differences between the zones analyzed (*p* > 0.05).

**Table 1:** Descriptive statistics of metals and physicochemical parameters by site type. Results are presented as median and interquartile range (Q1–Q3) due to skewed distributions and zero inflation observed for some variables. Negative COD values reported by the laboratory were treated as non-quantifiable and excluded from quantitative analyses

Site type	***n***	Cu	Mn	Zn	Fe	pH	DO	Turbidity	BOD	COD
Rural	14	5.92 [4.42–8.30]	145.58 [118.11–193.85]	1.96 [1.80–2.45]	5.89 [4.26–7.22]	6.95 [6.85–7.10]	8.98 [8.75–9.79]	10.50 [8.38–15.30]	0.00 [0.00–3.75]	6.00 [4.00–27.00]
Urban	14	30.42 [19.56–32.62]	225.56 [173.70–352.09]	17.70 [8.14–24.55]	15.94 [9.95–24.10]	6.78 [6.68–6.79]	8.78 [8.42–12.27]	17.06 [13.58–19.80]	0.00 [0.00–3.00]	7.00 [7.00–10.00]
Intermediate	7	17.66 [14.07–25.33]	194.56 [183.31–204.85]	13.31 [11.14–35.74]	40.70 [24.70–50.98]	6.86 [6.82–6.92]	8.63 [8.29–11.05]	13.40 [11.57–17.80]	0.00 [0.00–3.00]	13.50 [8.50–20.75]

The difference between rural and urban areas can be attributed to the influence of land use on metal concentrations in the Rio do Campo watershed. The higher concentrations of Cu, Mn, Zn, and Fe in the urban area reflect the combined effects of processes inherent to this environment, including increased impermeable surfaces, greater stormwater runoff transporting metallic particles, and higher population density. This spatial differentiation reflects the cumulative effects of land-use intensification along the watershed. This pattern is common in small rural–urban basins, where Zn and Cu are among the metals most responsive to urban inputs (Dibofori-Orji et al., 2019), while Fe and Mn tend to respond simultaneously to geology and increases in suspended sediments (Bhering et al,  2007).

Although P5 was not included in the paired statistical analysis, its values, similar to those of the urban points, indicate that the intermediate reach functions as a transition zone, influenced by urban occupation and its downstream position relative to a WTP. The observed difference in turbidity reinforces this pattern, as urban environments exhibit greater hydrosedimentary instability and higher availability of particles that act as vectors for metals (Zohar et al., 2017).

The opposite behavior of pH, with higher values in the rural zone, suggests lower inputs of organic matter and reduced acidification, conditions known to influence metal solubility and transport (Tessier & Turner, 1995).

Thus, the comparison between zones demonstrates that metal dynamics in the basin are modulated both by direct anthropogenic factors, most evident in urban environments, and by natural conditions that influence the release and transport of these elements. This interpretation aligns with similar assessments conducted in the Rio do Campo watershed by Manari, (2023), who identified converging issues in the middle course, and by França et al. (2022), whose morphometric and hydrosedimentary analyses of the Km 119 River indicate a progressive intensification of impacts along the rural–urban gradient.

### Comparison among sampling points

Table 2 presents the medians and interquartile ranges (Q1–Q3) of the variables analyzed. P2, P3, and P5 showed the highest values of Cu, Zn, and Fe, whereas P1 and P4 consistently exhibited the lowest concentrations throughout the sampling months.

**Table 2:** Descriptive statistics of metals and physicochemical parameters by sampling point. Values are expressed as median and interquartile range (Q1–Q3). This approach was adopted due to non-normal distributions and the absence of laboratory-reported detection or quantification limits

Sampling point	Site type	***n***	Cu	Mn	Zn	Fe	pH	DO	Turbidity	BOD	COD
P1	Rural	7	5.96 [4.90–9.75]	188.69 [146.15–209.16]	2.45 [1.90–5.64]	5.07 [4.41–6.73]	7.07 [7.01–7.13]	8.99 [8.83–10.18]	13.50 [10.15–16.20]	0.00 [0.00–2.50]	6.00 [4.50–8.00]
P2	Urban	7	30.31 [21.21–31.74]	199.92 [153.39–233.50]	17.65 [8.25–19.64]	22.39 [18.29–30.25]	6.77 [6.68–6.99]	8.84 [8.51–12.42]	19.50 [14.44–20.70]	0.00 [0.00–2.50]	7.50 [2.50–8.75]
P3	Urban	7	31.48 [17.53–33.52]	295.99 [217.20–375.38]	17.74 [8.38–26.34]	10.54 [9.12–13.73]	6.78 [6.71–6.79]	8.71 [8.49–11.82]	15.90 [13.60–18.98]	0.00 [0.00–5.00]	7.00 [7.00–10.50]
P4	Rural	7	4.99 [4.60–6.50]	126.58 [96.57–145.58]	1.93 [1.57–2.15]	6.34 [4.92–7.02]	6.86 [6.81–6.91]	8.96 [8.69–9.50]	8.50 [8.02–11.00]	0.00 [0.00–4.50]	15.50 [2.50–27.00]
P5	Intermediate	7	17.66 [14.07–25.33]	194.56 [183.31–204.85]	13.31 [11.14–35.74]	40.70 [24.70–50.98]	6.86 [6.82–6.92]	8.63 [8.29–11.05]	13.40 [11.57–17.80]	0.00 [0.00–3.00]	13.50 [8.50–20.75]

Significant differences were observed among points for Cu (*p* = 0.00296), Zn (*p* = 0.00518), Fe (*p* = 0.00982), pH (*p* = 0.0461), and turbidity (*p* = 0.00518), according to the Friedman test with Holm correction. For metals, P2, P3, and P5 presented higher values than P1 and P4, indicating greater anthropogenic influence at these locations. Urbanized areas tend to accumulate more metals due to increased surface runoff, soil sealing, and the input of contaminated particles (Zohar et al., 2017).

For Mn, only the comparison between P3 and P4 was significant (Nemenyi post hoc test, *p* = 0.0035). This behavior may be associated with the natural transport of sediments derived from the region’s soils, which are rich in iron and manganese. The natural mobilization of soil materials and the transport of fine particles contribute to metal enrichment in sediments, as discussed by Huang et al. (2020), who identified the influence of geogenic processes and fine sediment fractions on metal distribution.

pH was higher at P1, indicating lower organic matter input and reduced acidification in preserved stretches. The absence of significant differences in DO (*p* = 0.642), BOD (*p* = 0.642), and COD (*p* = 0.263) among points suggests that the contrasts observed in metal distribution are more closely related to hydrosedimentary dynamics and the physical characteristics of the surrounding environment than to processes of organic pollution (Poleto & Martinez, 2011).

The comparison among points demonstrates that the basin exhibits a gradient of modification, in which P2, P3, and P5 represent stretches more affected by urban and transitional pressures, whereas P1 and P4 reflect conditions of lower anthropogenic interference. The spatial variation detected highlights the watershed’s sensitivity to land-use changes, such as increased turbidity, suspended solids, and metals in urbanized areas due to surface runoff and sediment input (Villwock & Crispim, 2019; Da Silva et al., 2020). This pattern evidences the increasing fragility of the Rio do Campo watershed in sections where riparian vegetation is reduced or where anthropogenic use intensifies.

### Correlation among variables

**Fig. 2:**
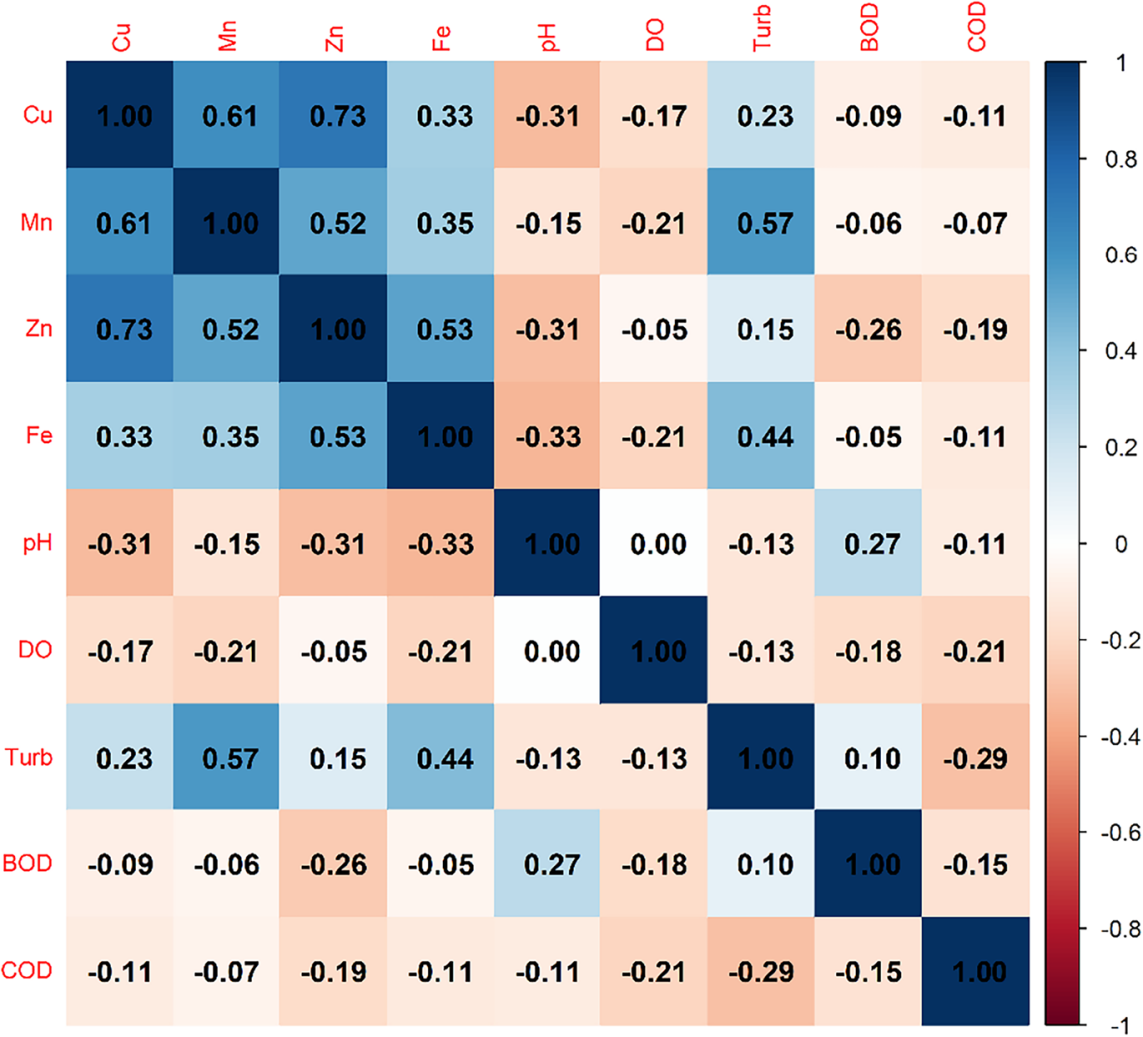
Pearson correlation matrix among metals (Cu, Mn, Zn, Fe) and physicochemical parameters (pH, dissolved oxygen, turbidity, biochemical oxygen demand, and chemical oxygen demand), calculated using complete observations. Color intensity and direction indicate the strength and sign of correlations

The correlation matrix revealed strong and coherent associations between environmental variables and metal concentrations (Fig. 2).

These associations are consistent with co-mobilization pathways driven by lithology and particle transport. The strongest correlations occurred between Cu–Zn (*r* = 0.73) and Zn–Fe (*r* = 0.53), indicating joint behavior and a possible shared origin or similar transport processes.

The positive correlation between Mn and turbidity (*r* = 0.57) suggests that increased suspended particle loads are related to the transport of Mn. This behavior may be reinforced both by local geology (Red Latosols) and by intensified sediment transport in urban areas, resulting from enhanced erosive processes in impacted stretches (Nascimento et al., 2018).

pH showed moderate negative correlations with Cu (*r* = − 0.31), Zn (*r* = − 0.31), and Fe (*r* = − 0.33), consistent with higher metal solubility under more acidic conditions (Belo et al., 2010), which were observed mainly in urban and intermediate stretches. DO also showed weak negative correlations with metals (*r* ranging from − 0.05 to − 0.21), reflecting the sensitivity of dissolved oxygen to hydrological conditions and particulate material, but without indicating direct influence from organic load, as demonstrated by its low correlation with BOD (*r* = − 0.18) and COD (*r* = − 0.21).

**Fig. 3:**
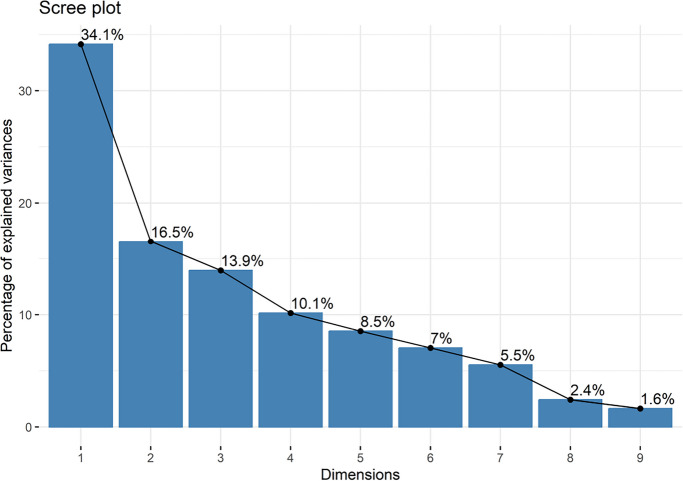
Scree plot showing the percentage of variance explained by each principal component derived from the PCA. The first principal component (PC1) explains 34.1% of the total variance, while the first three components together account for 64.6% of the cumulative variance

The PCA reinforces these patterns, explaining 64.6% of the total variance in the first three components (Fig. 3).

The eigenvalues, factor loadings, and explained variance are presented in Tables 3 and 4.

**Table 3:** Factor loadings of the first three principal components (PC1–PC3) obtained from the PCA of environmental variables and metal concentrations. Positive and negative loadings indicate the direction and strength of each variable’s contribution to the components

Variable	PC1	PC2	PC3
Cu	0.457	0.086	− 0.011
Mn	0.448	− 0.157	− 0.082
Zn	0.463	0.201	0.148
Fe	0.400	− 0.043	− 0.061
pH	− 0.260	− 0.413	0.049
DO	− 0.137	0.213	0.719
Turbidity	0.328	− 0.426	0.065
BOD	− 0.097	− 0.616	− 0.160
COD	− 0.112	0.385	− 0.647

**Table 4:** Eigenvalues, percentage of variance explained, and cumulative variance for the first three principal components derived from the PCA

Component	Eigenvalue	Variance (%)	Cumulative (%)
PC1	3.07	34.14	34.14
PC2	1.49	16.55	50.69
PC3	1.25	13.94	64.63

PC1 (34.1%) reflected a metal gradient, with high loadings for Cu, Mn, Zn, Fe, and turbidity, while pH and DO loaded negatively. This component represents the main environmental axis of the watershed, indicating that metal distribution reflects both urban inputs and the mobilization of particles derived from local lithology.

PC2 (16.5%) was driven primarily by dissolved oxygen, with secondary contributions from COD, capturing variations in water physicochemical conditions. PC3 (13.9%) was characterized mainly by contributions from Zn and Fe, reflecting additional variability in metal-associated processes.

**Fig. 4:**
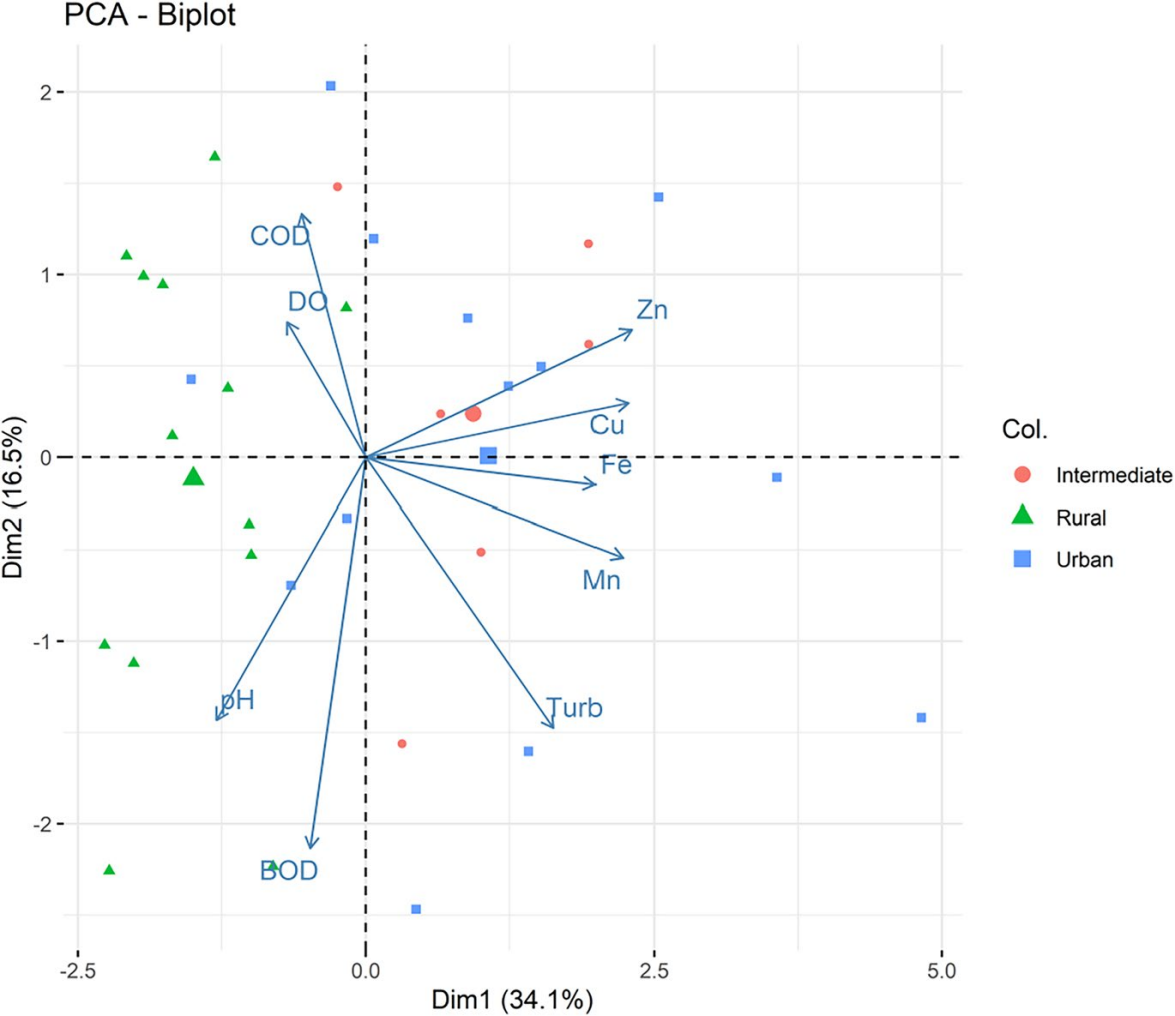
Principal component analysis (PCA) biplot based on complete-case standardized data (centered and scaled). Arrows represent the loadings of metals (Cu, Mn, Zn, Fe) and physicochemical parameters (pH, dissolved oxygen, turbidity, biochemical oxygen demand, and chemical oxygen demand), while points represent sampling observations classified according to land-use categories (rural, urban, and intermediate). The first two principal components explain 34.1% (PC1) and 16.5% (PC2) of the total variance, respectively

The analyses also revealed overlap among samples from the rural, urban, and intermediate categories (Fig. 4). This pattern indicates that the fluvial system exhibits a continuous gradient rather than discrete groups.

Overall, the PCA revealed two major gradients: metal and particle-related processes (PC1) and water physicochemical conditions (PC2). The integrated results from the correlations and PCA indicate that metal dynamics in the watershed are controlled by multiple interconnected processes, in which anthropogenic contributions overlap with, but do not override, natural characteristics. The spatial overlap among zones reinforces that the Rio do Campo responds continuously to environmental pressures, with particle transport playing a central role in metal distribution, consistent with environmental and hydrosedimentary studies conducted in the same region.

## Conclusion

The study revealed that the distribution of Cu, Mn, Zn, and Fe in the Rio do Campo watershed is modulated by land use and land cover, resulting in consistent spatial patterns along the rural–urban gradient. The highest metal concentrations were recorded at the urban points (P2 and P3) and in the intermediate zone (P5), indicating that the effects of urbanization propagate downstream. In contrast, P1 and P4 exhibited lower and more stable values, characteristic of areas with reduced anthropogenic influence.

The observed patterns confirm that hydrosedimentary processes, particularly elevated turbidity and associated particulate transport, exert a stronger influence on metal mobility than variations in organic load. The relationship between Mn and turbidity, as well as the Cu–Zn–Fe associations, points to a shared origin and dynamics linked both to local lithology and to increased sediment transport caused by soil sealing and changes in vegetation cover. The pH response reinforces this mechanism, indicating conditions more favorable to metal solubilization in urbanized stretches.

The PCA showed that the system does not operate in discrete rural, urban, or intermediate compartments. Instead, the watershed behaves as an interconnected environmental continuum in which pressures blend and intensify as they move downstream. The similarity between the intermediate point and the urban stretches summarizes this effect, aligning with regional studies that report a downstream expansion of impacts toward transitional areas.

Although all metal concentrations remained within legal limits, the detected spatial variability reveals a scenario of increasing environmental vulnerability. The similarity between the intermediate point and the urban stretches summarizes this effect, aligning with regional studies that have reported a downstream expansion of impacts toward transitional areas.

Thus, the study demonstrates that water quality in the Rio do Campo watershed results from a combination of natural factors, such as the lithology of Red Latosols, and anthropogenic pressures that accumulate along the fluvial gradient. The convergence among metals, turbidity, pH, and spatial patterns reinforces the need for management strategies that consider watershed connectivity, protection of riparian vegetation, and strengthened actions to control urban runoff and erosion. These elements are essential to mitigate the propagation of impacts and preserve the hydrological and ecological resilience of the system.

## Supplementary Information

Below is the link to the electronic supplementary material.

ESM 1 (DOCX 15.0 KB)

## Data Availability

No datasets were generated or analysed during the current study.
