# Supplementary material for: Influence of urban and agricultural land use on trace metal contamination in the Rio do Campo watershed, Paraná, Brazil
Source: Environ Monit Assess. 2026 Mar 22;198(4):340. doi: 10.1007/s10661-026-15150-2 (PMC13005772; doi:10.1007/s10661-026-15150-2)
Supplement: Supplementary file 1 — (DOCX 15.0 KB) [file 10661_2026_15150_MOESM1_ESM.docx]

**Supplementary Material**

Limits of Detection and Quantification (LOD/LOQ)

**Table 1**. Limits of Detection and Quantification for water samples.

| Analyte | Matrix | Method (SMWW) | LOD | LQ | Units | QA/QC Notes |
| --- | --- | --- | --- | --- | --- | --- |
| Iron (Fe) | Water | 3030E / 3111B |  | 0.191 mg/L | mg/L | Flame AAS; totals or dissolved per filtration/preservation |
| Manganese (Mn) | Water | 3030E / 3111B |  | 0.015 mg/L | mg/L | Flame AAS |
| Copper (Cu) | Water | 3030E / 3111B |  | 0.060 mg/L | mg/L | Flame AAS |
| Zinc (Zn) | Water | 3030E / 3111B |  | 0.067 mg/L | mg/L | Flame AAS |

**Table 2.** Limits of Detection and Quantification for sediment samples.

| Analyte | Matrix | Extraction/Method | LOD | LQ | Units | QA/QC Notes |
| --- | --- | --- | --- | --- | --- | --- |
| Manganese (Mn) | Sediment | B-HCl 0.05 mol/L |  |  | mg/kg (dry) |  |
| Copper (Cu) | Sediment | Mehlich 1 or B-HCl (specify) |  |  | mg/kg (dry) | Match to TEL/PEL comparison |
| Zinc (Zn) | Sediment | Mehlich 1 or B-HCl (specify) |  |  | mg/kg (dry) |  |

| **Metal / Parâmetro** | **Método Analítico (SMWW 24ª ed.)** | **Técnica** | **LQ (mg/L)** | **Referência Normativa*** |
| --- | --- | --- | --- | --- |
| **Cobre (Cu)** | 3030 E (preparação) / 3111 B (determinação) | EAA chama ar-acetileno | 0,060 | ABNT NBR ISO/IEC 17025 |
| **Zinco (Zn)** | 3030 E / 3111 B | EAA chama ar-acetileno | 0,067 | ABNT NBR ISO/IEC 17025 |
| **Ferro (Fe)** | 3030 E / 3111 B | EAA chama ar-acetileno | 0,191 | ABNT NBR ISO/IEC 17025 |
| **Manganês (Mn)** | 3030 E / 3111 B | EAA chama ar-acetileno | 0,015 | ABNT NBR ISO/IEC 17025 |
| **Alumínio (Al)** | 3500 Al B | Colorimétrico (Eriocromo Cianina R) | 0,020 | ABNT NBR ISO/IEC 17025 |
